# Evaluation of corn fermented protein on the fecal microbiome of cats

**DOI:** 10.1093/jas/skae268

**Published:** 2024-09-14

**Authors:** Logan R Kilburn-Kappeler, Tyler Doerksen, Andrea Lu, Rachel M Palinski, Nanyan Lu, Charles G Aldrich

**Affiliations:** Department of Grain Science and Industry, Kansas State University, Manhattan, KS 66506, USA; Veterinary Diagnostic Laboratory, College of Veterinary Medicine, Kansas State University, Manhattan, KS 66506, USA; Veterinary Diagnostic Laboratory, College of Veterinary Medicine, Kansas State University, Manhattan, KS 66506, USA; Veterinary Diagnostic Laboratory, College of Veterinary Medicine, Kansas State University, Manhattan, KS 66506, USA; Department of Diagnostic Medicine/Pathobiology, College of Veterinary Medicine, Kansas State University, Manhattan, KS 66506, USA; Bioinformatics Center, Kansas State University, Manhattan, KS 66506, USA; Department of Grain Science and Industry, Kansas State University, Manhattan, KS 66506, USA

**Keywords:** 16S rRNA gene sequencing, feline, yeast

## Abstract

Co-products from the ethanol industry, such as distillers dried grains with solubles (DDGS), can provide alternative protein sources for pet food. Corn fermented protein (CFP) is produced using postfermentation technology to split the protein and yeast from fiber prior to drying. This results in a higher protein ingredient compared to DDGS, increasing its appeal for pet food. In addition, the substantial yeast component, at approximately 20% to 25%, may promote gut health through modulation of the microbiome and the production of short-chain fatty acids. Therefore, the objective of this study was to determine the effect of CFP on the fecal microbiome of cats. The 4 experimental diets included a control with no yeast (T1) and diets containing either 3.5% brewer’s dried yeast (T2), 2.5% brewer’s dried yeast plus 17.5% DDGS (T3), or 17.5% CFP (T4). All diets except T1 were formulated to contain 3.5% yeast. Diets were fed to adult cats (*n* = 11) in an incomplete 4 × 4 replicated Latin square design. Cats were adapted to diet for 9 d followed by a 5-d total fecal collection. During each collection period, fresh fecal samples from each cat were collected and stored at −80 °C until analysis. Fresh fecal samples (*n* = 44) were analyzed by 16S rRNA gene sequencing. Raw sequences were processed through Mothur (v.1.44.1). Community diversity was evaluated in R (v4.0.3). Relative abundance was analyzed within the 50 most abundant operational taxonomic unitsusing a mixed model of SAS (v9.4, SAS Institute, Inc., Cary, NC). Diet was the fixed effect and cat and period were random effects. Results were considered significant at *P* < 0.05. Alpha-diversity indices (Observed, Chao1, Shannon, Simpson) and beta-diversity metric (principal coordinate analysis) were similar for all treatments. Predominant phyla were Firmicutes (66%), Bacteroidetes (25%), Actinobacteria (8%), Proteobacteria (0.64%), and Desulfobacteria (0.54%). The relative abundance of Firmicutes and Actinobacteria was lower (*P *< 0.05) for T3 compared to T4 and T2, respectively. On a more specific phylogenic level, 17 genera resulted in differences (*P* < 0.05) among dietary treatments. Overall, this data indicates that compared to traditional yeast and distillers dried grains, CFP did not alter the overall diversity of the fecal microbiome of healthy adult cats over a 14-d period.

## Teaser

The overall diversity of the fecal microbiome of cats was comparable when fed diets containing either soybean meal, brewer’s dried yeast, a combination of brewer’s dried yeast and distillers dried grains with solubles, or corn fermented protein. However, the relative abundance of taxonomic classifications shifted based on dietary substrate available for microbial fermentation.

## Introduction

Even though cats are strict carnivores with a short colon and nonfunctional cecum, they have considerable microbial activity in the hindgut (Rochus et al., 2014). Therefore, the addition of prebiotics to feline diets can benefit intestinal and host health (Barry et al., 2010; De Godoy et al., 2013; Garcia-Mazcorro and Minamoto, 2013; Felssner et al., 2016). Yeast derivatives, such as beta-glucans and mannan oligosaccharides (MOS), are used as prebiotics in the pet food industry. These derivatives have been reported to lower intestinal pH, promote microbial diversity, and reduce protein fermentation products (Barry et al., 2010; Kanakupt et al., 2011).

The composition of the microbiome is highly influenced by diet. Therefore, a change in dietary substrate can shift the microbial population to promote host health. For example, one of the main benefits of intestinal microbiota modulation is increased production of short-chain fatty acids (**SCFA**), which can be achieved by increasing dietary carbohydrate content (Mills et al., 1999; Wong et al., 2006; Barko et al., 2018). However, dietary substrate can also result in undesirable compounds such as ammonia and phenols from microbial fermentation of protein (De Preter et al., 2010; Ephraim et al., 2020; Jackson et al., 2020). Therefore, a nutrient balance is required not only to meet animal requirements and consumer demands but also to support intestinal health.

Corn fermented protein (**CFP**) could help to achieve this nutrient balance in pet food by providing protein as well as a substrate for microbial fermentation. CFP, a co-product of ethanol production, is produced using postfermentation technology to split the protein and yeast from fiber prior to drying. This results in a higher protein ingredient compared to traditional distillers dried grains, increasing its appeal for pet food among consumers. In addition, the substantial yeast component, at approximately 20% to 25%, may promote gut health. Previous studies have evaluated the effect of high protein distillers dried grains (**HPDDG**) on the fecal microbiome of dogs, indicating potential benefits for intestinal functionality (Kaelle et al., 2023; Kilburn-Kappeler et al., 2023b). For example, Kaelle et al. (2023) reported a linear increase in the number of operational taxonomic units, a quadratic effect for the Shannon index, and a trend for a linear increase in the Chao1 index with increased inclusion of HPDDG. Therefore, the objective of this study was to investigate the effects of CFP compared to traditional ingredients on the fecal microbiome of cats. It was hypothesized that the overall diversity of the microbiome would be comparable among dietary treatments however, there would be differences among taxonomic classifications due to the type of substrate reaching the large intestine.

## Materials and Methods

The feeding trial was conducted at Kansas State University Veterinary Medicine Complex (Coles Hall) under the Institutional Animal Care and Use Committee (IACUC) #4348 protocol.

### Diet formulation, production, and nutrient composition

Dietary treatments consisted of a control diet containing 15% soybean meal (T1) and experimental diets containing either 3.5% brewer’s dried yeast (T2), 2.5% brewer’s dried yeast plus 17.5% distillers dried grains with solubles (**DDGS**; T3), or 17.5% CFP (T4). It was assumed that CFP had 20% yeast and DDGS had 5.7% yeast; therefore, all treatments, except T1, were formulated to contain 3.5% yeast. It is important to note that the composition of yeast in ethanol co-products is not well established because of a lack of standardized analytical methods to determine the dead yeast in these ingredients. Published estimates of the contributions of yeast to DDGS biomass are extremely variable (Shurson, 2018). Therefore, the concentration of yeast in CFP and DDGS used in this study was an estimate provided by the ingredient sponsor (POET Bioproducts, Sioux Falls, SD) and not directly analyzed. The amount of yeast in the dietary treatments was then calculated based on these estimates. The formulated diets met the AAFCO nutritional requirements for adult cats (AAFCO, 2019). The amount of corn, chicken meal, and chicken fat was adjusted between base rations to maintain nutrient composition among dietary treatments and result in a complete formula (100%). The first base ration was used for T1 and T4, and included all dry ingredients, except for the soybean meal (**SBM**, Fairview Mills, Seneca, KS), CFP (POET Bioproducts), corn starch (Fairview Mills), corn gluten meal (Fairview Mills), and titanium dioxide (Fairview Mills). The second base ration was used for T2 and T3, and contained all dry ingredients except for SBM, DDGS (Fairview Mills), corn starch, corn gluten meal, brewer’s dried yeast (**BDY**; Fairview Mills), and titanium dioxide. Soybean meal, corn gluten meal, and/or corn starch were added to T1, T2, and T4 to create similar nutrient profiles among all dietary treatments and to balance a 20% inclusion of experimental ingredients compared to T3 (Table 1). Of note, diets were formulated and produced prior to chemical analysis of each specific ingredient used. Therefore, the final diets fluctuated in nutrient and calorie content.

**Table 1.** Ingredient composition of feline diets containing yeast and ethanol co-products on an as-is basis

	Treatment^1^			
Ingredient, %	T1	T2	T3	T4
Corn	34.6	30.0	30.0	34.6
Chicken meal	30.0	35.0	35.0	30.0
Soybean meal	15.0	8.0	—	—
Distillers dried grains with solubles	—	—	17.5	—
Corn fermented protein	—	—	—	17.5
Brewer’s dried yeast	—	3.5	2.5	—
Corn starch	—	6.5	—	2.5
Corn gluten meal	5.0	2.0	—	—
Chicken fat	6.0	5.6	5.6	6.0
Other^2^	9.4	9.4	9.4	9.4

^1^T1 = control; T2 = brewer’s dried yeast; T3 = brewer’s dried yeast and DDGS; T4 = CFP.

^2^Other ingredients: beet pulp (4.0%), fish meal (3.0%), flavor (1.0%), titanium dioxide (0.4%), salt (0.25%), potassium chloride (0.25%), vitamin and mineral premix (0.25%), choline chloride (0.20%), natural antioxidant (0.05%).

Each diet was mixed and produced using a single screw extruder (model E525, Extru-Tech, Manhattan, KS). The cool and dry product was packaged in laminated bags and transferred to the laboratory at Kansas State University to be coated. Kibble was coated with chicken fat protected with natural antioxidants (Nutrios, Springfield, MO) and a dry powdered flavor designed for cats (AFB International, St. Charles, MO). Coated diets were stored in polylined Kraft paper bags until fed.

Diets were analyzed in duplicate for moisture (AOAC 930.15), ash (AOAC 942.05), fat by acid hydrolysis and hexane extraction (AOAC 960.39), gross energy (Parr 6200 Calorimeter, Parr Instrument Company, Moline, IL), and total dietary fiber (AOAC 991.43). Crude protein was determined by Dumas combustion (AOAC 990.03) using a nitrogen analyzer (FP928, LECO Corporation, Saint Joseph, MI). Nutrient composition of dietary treatments is presented in Table 2.

**Table 2.** Analyzed chemical composition of feline diets containing yeast and ethanol co-products on a dry matter basis

	Treatment^1^			
Nutrient	T1	T2	T3	T4
Dry matter, %	95.03	95.71	94.38	95.50
Organic matter, %	90.03	90.17	90.31	91.49
Ash, %	9.97	9.83	9.69	8.51
Crude protein, %	41.59	40.58	38.50	37.22
Fat, %	12.80	13.32	14.45	13.40
Total dietary fiber, %	13.66	13.19	18.46	15.05
Insoluble dietary fiber, %	10.09	10.04	14.34	12.40
Soluble dietary fiber, %	3.67	3.14	4.13	2.64
Gross energy, kcal/kg	4951.82	4997.76	5065.36	5001.77

^1^T1 = control; T2 = brewer’s dried yeast; T3 = brewer’s dried yeast and DDGS; T4 = CFP.

### Feeding trial

Eleven healthy adult (3.1 ± 1.7 years) American shorthair cats (10 males and 1 female) were enrolled in an incomplete 4 × 4 triplicated Latin square design. Each of the 4 periods was composed of 9 d for diet adaptation followed by 5 d for fecal collection. A recent study by Lin et al. (2022) reported that the microbiome of dogs stabilized 6 d after dietary intervention. This can be used as a prediction for cats, indicating that the 9-d adaptation period in the current study was sufficient. Cats had an average body weight of 5.6 ± 1.7 kg, and food allowance was controlled to maintain their weight throughout the study. The daily metabolizable energy requirement was calculated for lean cats with 100*BW_kg_^0.67^ (NRC, 2006). The cats received 2 feedings per day at 0800 and 1700 hours with access to food for 1 h and water ad libitum. During the adaptation period, the cats were group-housed but fed individually. Whereas in the collection period, the cats were individually housed in stainless steel cages. During each collection period, a fresh fecal sample (within 15 min of defecation) from each cat was collected using a sterile Whirl-Pak bag, and 2 g aliquots were transferred with a spatula into plastic microcentrifuge tubes and stored at −80 °C for DNA extraction.

### Fecal DNA extraction and sequencing

The DNA was extracted from 200 mg of each stool sample (*n* = 44) using a QIAamp Power Fecal Pro DNA Kit (Qiagen, Hilden, Germany) and Qiacube Connect (Qiagen) in accordance with the manufacturer’s instructions (Handbook 02/2020). A nanodrop (NanoDrop 2000, Thermo Scientific, Waltham, MA) was used for quality control of nucleic acid purity. Extractions were quantified on a Qubit fluorometer (Qubit 4.0, Invitrogen by Life Technologies, Carlsbad, CA). The 16S V3/V4 region was amplified using the Illumina 16S Metagenomic Sequencing Library Preparation protocol (Illumina, Inc., San Diego, CA) as specified by the manufacturer. The size and quality of libraries and the pool were assessed with a 2100 Bioanalyzer (Agilent, Santa Clara, CA). Libraries were run on an Illumina MiSeq system using 300 × 2 v3 paired end chemistry.

### Data analysis

Raw reads were trimmed for quality using CLC Genomics Workbench (Qiagen, v11.0.1) and then imported into Mothur (v1.44.1) for further analysis (Schloss et al., 2009). The unique 16S reads were aligned to reference sequences from the SILVA rRNA database (Release 138) for closed-reference operational taxonomic unit (OTU) assignment (Yilmaz et al., 2014). Near-identical sequences were merged using VSEARCH v2.15.1 (Rognes et al., 2016).

Bacterial counts were log transformed using the log10 command and evaluated using the phyloseq package (McMurdie and Holmes, 2013) in R (v4.0.3, R Core Team, 2019). Beta-diversity was determined by a principal coordinate analysis (**PCoA**). Alpha-diversity was assessed using observed unique sequences, Chao1, Shannon, and Simpson indices. One sample (T4, cat 8, period 4) was considered an outlier and removed from the data. To determine differences among treatment means, diversity measures were analyzed in a one-way ANOVA in R. Relative abundance data were analyzed within the 50 most abundant OTU using a GLIMMIX procedure in SAS (v9.4, SAS Institute, Inc., Cary, NC). Treatment was considered a fixed effect and cat and period as random effects. Tukey’s post hoc test was applied for the least-squares means separation, with significance considered at *P *< 0.05.

## Results and Discussion

### Beta and alpha diversity

**Figure 1.**
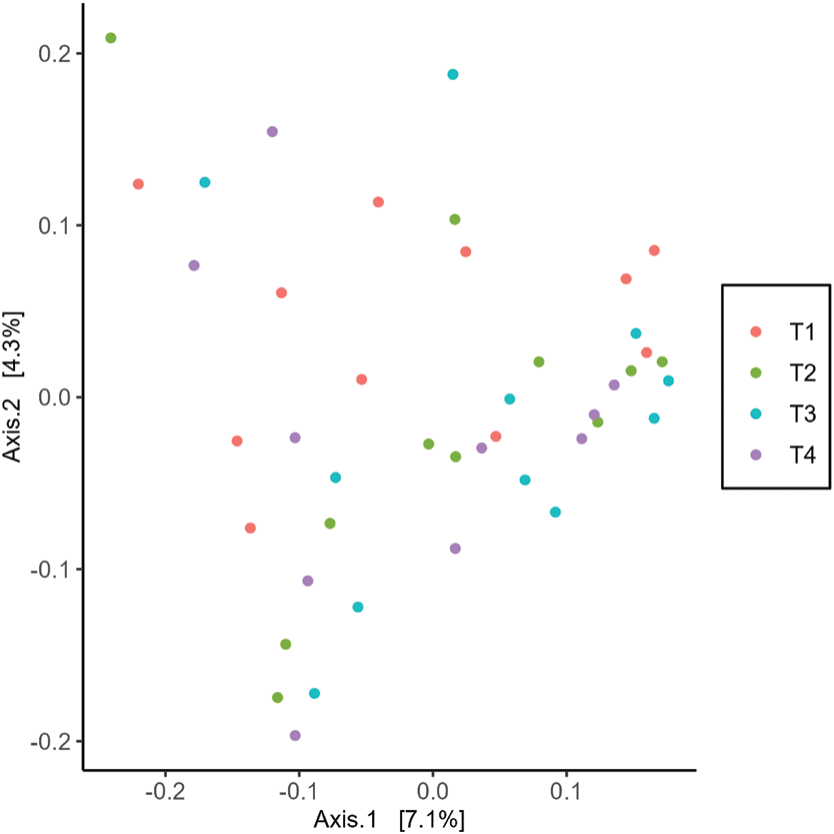
PCoA explaining 7.1% and 4.3% of the variability in operational taxonomic units of Bray–Curtis UniFrac distances for fecal samples from cats fed dietary treatments. Treatments: T1 = control; T2 = brewer’s dried yeast; T3 = brewer’s dried yeast and DDGS; T4 = CFP.

Beta diversity is a representation of the entire microbial community. PCoA plots relationships on a 2-dimensional scatterplot, with the axes representing fractions of variability. Each point on the scatterplot represents a single sample, and the distance between points represents how compositionally different the samples are from each other (Koleff et al., 2003). Consequently, PCoA plots can be interpreted as samples with high similarity appearing as clusters, and samples with high dissimilarity appearing randomly dispersed (Goodrich et al., 2014). Therefore, the lack of apparent clustering by treatment groups in the current study indicates that beta diversity was not altered by dietary treatments (Figure 1).

**Figure 2.**
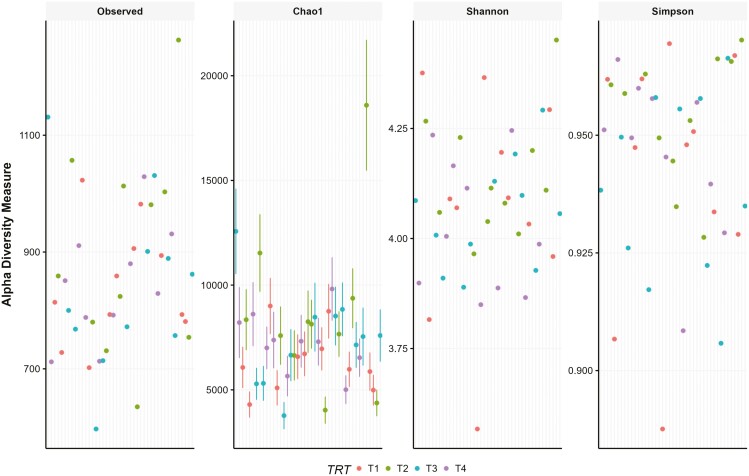
Alpha diversity measures for fecal samples from cats fed dietary treatments. Treatments: T1 = control; T2 = brewer’s dried yeast; T3 = brewer’s dried yeast and DDGS; T4 = CFP.

Alpha diversity represents the within-sample variation for each cat’s microbiome. There are several computed indices that have been proposed to help describe the number of species in a sample (richness) and how dominant or rare each species is to the others (diversity; Wagner et al., 2018). For the current study, the Observed index (number of OTU), Chao1 index (species richness), Shannon index (species diversity), and Simpson index (species evenness) were evaluated. Alpha-diversity indices were similar (*P* > 0.05) for all treatments (Figure 2), indicating that dietary treatments did not influence the species richness or diversity within the samples.

A healthy microbiome is characterized by high species richness and diversity with a reduction often associated with disease. For example, cats with chronic enteropathy had decreased alpha diversity compared to healthy cats (Marsilio et al., 2019; Kathrani et al., 2022). In the current research, the overall diversity of the microbiome was maintained when cats were fed BDY, BDY + DDGS, or CFP compared to the control containing SBM suggesting the experimental treatments did not hamper the ecosystem of the colon. In a similar fashion, beta and alpha diversity were comparable among dietary treatments when fed to dogs (Kilburn-Kappeler et al., 2023b).

### Phyla relative abundance

Most studies have reported that the microbiome of healthy cats is dominated by Firmicutes, followed by Proteobacteria, Actinobacteria, Bacteroidetes, and Fusobacteria (Ritchie et al., 2008; Handl et al., 2011; Eshar et al., 2019; Ganz et al., 2022). The most abundant phyla among all samples in the current study were Firmicutes at 66%, Bacteroidetes at 25%, Actinobacteria at 8%, Proteobacteria at 0.64%, and Desulfobacteria at 0.54% (Table 3). The predominant phylum in the current study is similar to the previous studies in healthy cats except for Desulfobacteria which were observed instead of Fusobacteria.

**Table 3.** Relative abundance of bacterial phyla among the 50 most abundant operational taxonomic units in fecal samples from cats fed dietary treatments

	Treatment^1^					
Phylum, %	T1	T2	T3	T4	SEM	*P*-value
Firmicutes	66.07^a,b^	66.71^a,b^	60.75^b^	69.39^a^	2.875	0.0423
Bacteroidetes	23.37	22.70	31.32	22.06	3.321	0.0294^*^
Actinobacteria	9.15^a,b^	9.67^a^	6.50^b^	7.61^a,b^	1.183	0.0375
Proteobacteria	0.56	0.53	0.84	0.64	0.914	0.1756
Desulfobacteria	0.71	0.39	0.72	0.33	0.182	0.0755

^1^T1 = control; T2 = brewer’s dried yeast; T3 = brewer’s dried yeast and DDGS; T4 = CFP.

^a,b^Means within a row lacking a common superscript letter are different (*P* < 0.05).

^*^Tukey adjustment eroded the difference (*P* > 0.05) among treatment means.

The relative abundance of Firmicutes for T3 at 61% was lower (*P* < 0.05) than that of T4 at 69%, with T1 and T2 intermediate at 66% and 67%, respectively. The relative abundance of Actinobacteria was lower (*P* < 0.05) for T3 at 7% compared to T2 at 10%, with T1 and T4 intermediate at 9% and 8%, respectively. The comparison of the relative abundance of Bacteroidetes among dietary treatments resulted in a significant *P*-value, however, the Tukey adjustment eroded this difference (*P* > 0.05) among treatment means. Numerically, the relative abundance of Bacteroidetes was greater for T3 at 31% compared to T1 at 23%, T2 at 23%, and T4 at 22%. The relative abundance of Proteobacteria was also numerically greater for T3 at 0.8% compared to T1, T2, and T4 at 0.6%, 0.5%, and 0.6%, respectively. The relative abundance of Desulfobacteria was 0.7%, 0.4%, 0.7%, and 0.3% for T1, T2, T3, and T4, respectively.

The microbiome is responsive to nutrients rather than individual dietary ingredients (Pilla and Suchodolski, 2021). Therefore, the differences in phyla relative abundance among dietary treatments are likely due to the differences in nutrient composition, specifically fiber and protein. For example, dietary fiber has been reported to increase Firmicutes in dogs (Middelbos et al., 2010; Beloshapka et al., 2013; Panasevich et al., 2015). Therefore, the decrease in Firmicutes for T3 was surprising as T3 contained the greatest amount of fiber among dietary treatments. In addition, the differences in Actinobacteria may be due to the type of fiber present in dietary treatments (Barry et al., 2012). Furthermore, Hooda et al. (2013) reported a decrease in Actinobacteria in kittens fed a high protein/low carbohydrate diet compared to a moderate protein/moderate carbohydrate diet. Therefore, it could be expected that diets with higher protein content would result in a lower relative abundance of Actinobacteria when fed to cats. However, the opposite response was observed in the current study. Of note, the relative abundance of Firmicutes, Bacteroidetes, and Actinobacteria was not impacted by dietary treatments when fed to dogs (Kilburn-Kappeler et al., 2023b). The variable results among studies could be due to differences in dietary matrices, animal population, environment, and/or specific taxa (e.g., genera) present in each phylum.

### Genera relative abundance

When compared on a more specific phylogenic level, 17 genera out of the 50 most abundant OTU within samples resulted in significant differences among dietary treatments (Table 4). The relative abundance of *Acidaminococcus* was lower (*P* < 0.05) for T3 at 0.4% compared to T1 at 1.3% with T2 and T4 intermediate at 0.7% and 1.3%, respectively. The relative abundance of *Allisonella* was highest (*P* < 0.05) for T4 at 0.5% and lowest (*P* < 0.05) for T1 and T2 at 0.3%, T3 was intermediate at 0.4%. The relative abundance of *Bifidobacterium* was lower (*P* < 0.05) for T3 and T4 at 0.4% and 0.8%, respectively, compared to T1 and T2 at 2.6% and 2.4%, respectively. The relative abundance of *Blautia* was greater (*P* < 0.05) for T2 at 13.7% compared to T3 at 10.8% and T4 at 10.5%, T1 was intermediate at 11.6%. *Catenibacterium* relative abundance was greatest (*P* < 0.05) for T4 at 5.4% compared to T1, T2, and T3 at 2.4%, 3.6%, and 2.9%, respectively. The relative abundance of *Dialister* was greater (*P* < 0.05) for T4 at 4.6% compared to T1 at 2.3% and T2 at 3.0%, T3 was intermediate at 4.1%. The lowest (*P* < 0.05) relative abundance of *Erysipelatoclostridium* was observed for T1 at 0.1% compared to T2 at 0.9%, T3 at 1.1%, and T4 at 1.4%. *Fusicatenibacter* relative abundance was greater (*P* < 0.05) for T1 and T4 at 0.9% and 1.0%, respectively, compared to T2 at 0.4% with T3 intermediate at 0.8%. The relative abundance of *Holdemanella* was greater (*P* < 0.05) for T3 at 10.1% and T4 at 11.1% compared to T1 at 6.0% with T2 intermediate at 8.7%. *Megamonas* relative abundance was greater (*P* < 0.05) for T1 at 2.0% compared to T3 at 0.8% with T2 and T4 intermediate at 1.5% and 0.9%, respectively. *Peptoclostridium* relative abundance was greater (*P* < 0.05) for T4 at 9.2% compared to T1 and T3 at 5.0% and 6.9%, respectively, T2 was intermediate at 7.3%. The relative abundance of *Peptococcus* was greater (*P* < 0.05) for T2 at 1.5% compared to T3 at 1.1% and T4 at 1.0%, T1 was intermediate at 1.2%. The relative abundance of *Prevotella 9* was greater (*P* < 0.05) for T3 at 23% compared to T2 at 14.8% with T1 and T4 intermediate at 16.7% and 15.0%, respectively. The relative abundance of *Solobacterium* was greater (*P* < 0.05) for T3 at 3.2% compared to T1 and T2 at 1.3% with T4 intermediate at 3.0%. *Streptococcus* relative abundance was greater (*P* < 0.05) for T1 at 8.5% compared to T3 at 1.6% and T4 at 1.2% with T2 intermediate at 4.2%. The relative abundance of *Subdoligranulum* was greater (*P* < 0.05) for T1 at 4.9% and T2 at 3.3% compared to T3 at 1.5% and T4 at 1.1%. The genera classified as *uncultured* was greatest (*P* < 0.05) for T3.

**Table 4.** Relative abundance of bacterial genera among the 50 most abundant operational taxonomic units in fecal samples from cats fed dietary treatments

	Treatment^1^					
Genus, %	T1	T2	T3	T4	SEM	*P*-value
*Acidaminococcus*	1.33^a^	0.67^a,b^	0.43^b^	1.25^a,b^	0.299	0.0132
*Allisonella*	0.25^c^	0.25^c^	0.39^b^	0.52^a^	0.043	<0.0001
*Alloprevotella*	2.20	2.58	1.93	1.71	0.408	0.1950
*Bacteroides*	4.32	4.39	4.54	4.37	0.810	0.9934
*Bifidobacterium*	2.57^a^	2.39^a^	0.41^b^	0.77^b^	0.567	0.0007
*Blautia*	11.63^a,b^	13.68^a^	10.79^b^	10.53^b^	1.019	0.018
*Catenibacterium*	2.37^b^	3.56^b^	2.86^b^	5.38^a^	0.497	<0.0001
*Catenisphaera*	0.58	1.13	1.79	0.66	0.615	0.1973
*Collinsella*	6.13	6.93	5.87	6.04	0.762	0.5168
*Coprococcus*	0.94	0.59	0.78	0.87	0.215	0.3973
*Desulfovibrio*	0.71	0.39	0.72	0.33	0.182	0.0755
*Dialister*	2.28^c^	3.03^b,c^	4.13^a,b^	4.64^a^	0.523	0.0005
*Erysipelatoclostridium*	0.10^b^	0.93^a^	1.11^a^	1.41^a^	0.192	<0.0001
*Faecalibacterium*	1.28	1.30	2.29	2.06	0.439	0.0550
*Fusicatenibacter*	0.93^a^	0.43^b^	0.78^a,b^	1.03^a^	0.166	0.0068
*Holdemanella*	5.97^b^	8.73^a,b^	10.11^a^	11.14^a^	1.164	0.0010
*Lachnospiraceae ge*	2.17	2.00	2.32	2.36	0.221	0.3707
*Lachnospiraceae unclassified*	1.98	2.52	1.99	2.19	0.576	0.2151
*Ligilactobacillus*	4.63	3.32	2.16	3.46	2.843	0.8517
*Megamonas*	2.04^a^	1.53^a,b^	0.80^b^	0.92^a,b^	0.455	0.0384
*Megasphaera*	3.52	3.07	1.75	3.20	0.524	0.0097^*^
*Negativibacillus*	1.28	1.31	1.36	0.90	0.191	0.1005
*Olsenella*	0.46	0.32	0.24	0.82	0.394	0.5068
*Peptoclostridium*	5.03^c^	7.31^a,b^	6.89^b,c^	9.18^a^	0.766	0.0002
*Peptococcus*	1.21^a,b^	1.52^a^	1.11^b^	1.04^b^	0.129	0.0047
*Peptostreptococcaceae unclassified*	1.72	1.06	0.65	1.18	0.591	0.3361
*Prevotella 9*	16.67^a,b^	14.83^b^	22.67^a^	15.03^a,b^	2.750	0.0250
*Solobacterium*	1.33^b^	1.31^b^	3.19^a^	2.96^a,b^	0.631	0.0050
*Streptococcus*	8.53^a^	4.24^a,b^	1.64^b^	1.19^b^	2.387	0.0173
*Subdoligranulum*	4.89^a^	3.31^a^	1.45^b^	1.08^b^	0.658	<0.0001
*Sutterella*	0.56	0.53	0.84	0.64	0.152	0.1756
*uncultured*	0.26^b^	0.92^b^	2.09^a^	0.98^b^	0.303	<0.0001

^1^T1 = control; T2 = brewer’s dried yeast; T3 = brewer’s dried yeast and DDGS; T4 = CFP.

^a,b,c^Means within a row lacking a common superscript letter are different (*P* < 0.05).

^*^Tukey adjustment eroded the difference (*P* > 0.05) among treatment means.

Ganz et al. (2022) reported the genera with the highest relative abundance in healthy pet cats were *Prevotella*, *Bacteroides*, *Collinsella*, *Catenibacterium*, *Blautia*, *Faecalibacterium*, and *Megasphaera*. Each of these genera was among the 50 most abundant OTU in the current study. Additional studies have also reported *Bacteroides* and *Prevotella* to be the most frequent genera observed in the microbiome of healthy cats (Johnston et al., 2001; Alessandri et al., 2020). Hooda et al. (2013) also reported that *Faecalibacterium* was common in the fecal microbiome of healthy kittens fed a range of diets.

Many of these genera are associated with the production of SCFA and branched chain fatty acids (**BCFA**) by fermentation of carbohydrates, protein, and fiber in the gastrointestinal tract (Mead, 1971; Levine et al., 2013; Butowski et al., 2019). For example, *Prevotella*, *Catenibacterium*, and *Megasphaera* digest glucose or lactateproducing propionate (Butowski et al., 2019). *Faecalibacterium* ferments carbohydrates producing acetate and can further convert this acetate to butyrate (Morrison and Preston, 2016). *Bacteroides*, *Blautia*, and *Collinsella* also ferment dietary carbohydrates to SCFA (Qin et al., 2019; Ziese and Suchodolski, 2021). Ganz et al. (2022) also reported prevalence of *Subdoligranulum* and *Fusicatenibacter* among healthy cats, which are positively associated with SCFA production (Takeshita et al., 2016; Van den Abbeele et al., 2020). The synthesis of SCFA is beneficial as butyrate is the preferred substrate used by the gut mucosa, propionate contributes to gluconeogenesis in the liver, and acetate is most concentrated in the blood (Morrison and Preston, 2016). Therefore, proportions of these genera and their end products can influence host health. However, the significant differences in relative abundance of genera did not impact the production of SCFA and BCFA among dietary treatments in the current study (Kilburn-Kappeler et al., 2023a).

*Bacteroides*, *Prevotella*, and *Faecalibacterium* have been reported to increase with increased dietary fiber content in dogs (Pilla and Suchodolski, 2021). Therefore, the increase in *Prevotella* for T3 could be explained by the increased dietary fiber content. In addition, Butowski et al. (2019) reported an increase in *Prevotella* with the addition of plant fiber to a raw diet fed to adult cats. An increase in *Blautia* was reported in kittens fed high protein/low carbohydrate diets compared to kittens fed medium protein/medium carbohydrate diets (Hooda et al., 2013). Therefore, the decrease in *Blautia* for T3 and T4 could be due to the decreased protein content in dietary treatments. In contrast, a decrease in *Bifidobacterium* was reported in kittens fed high protein/low carbohydrate diets (Vester et al., 2009; Hooda et al., 2013). Prebiotic fibers have also been reported to increase *Bifidobacterium* in cats (Bermingham et al., 2013; Hooda et al., 2013; Young et al., 2016). In addition, Beloshapka et al. (2013) reported an increase in *Bifidobacterium* in dogs fed diets containing yeast cell walls. The relative abundance of *Bifidobacterium* among dietary treatments in the current study indicates that different yeast sources may have varying effects on the fecal microbiome. The relative abundance of *Dialister* and *Acidaminococcus* among dietary treatments in the current study is interesting as decreased abundance was observed in kittens fed high protein/low carbohydrate diets (Hooda et al., 2013). Xu et al. (2021) reported a lower relative abundance of *Allisonella* and *Megamonas*, carbohydrate fermenters (Kieler et al., 2017; Butowski et al., 2019; Che et al., 2019), in dogs consuming a raw diet compared to a kibble diet. The relative abundance for both *Allisonella* and *Megamonas* was lower in T3 compared to T1, possibly indicating differences in dietary starch content. Schauf et al. (2018) reported a lower relative abundance of *Solobacterium* in dogs fed a high fat/low starch diet compared to a low fat/high starch diet. However, in the current study, the diet with the highest fat content (T3) resulted in a higher relative abundance of *Solobacterium*.

In contrast to the current study in cats, the relative abundance of *Bifidobacterium*, *Catenibacterium, Holdemanella*, *Peptococcus*, and *Prevotella* did not differ among dietary treatments when fed to dogs (Kilburn-Kappeler et al., 2023b). Furthermore, the relative abundance of *Collinsella* was impacted in dogs but not in cats fed dietary treatments. However, a similar effect in the relative abundance of *Blautia* was observed in both dogs and cats, with a significant decrease for T3 and T4. The effect of dietary treatments on the relative abundance of *Erysipelatoclostridium* was also similar for dogs and cats with the lowest abundance observed in T1. The relative abundance of *Peptoclostridium* was lower for T1 in both dogs and cats fed dietary treatments. In addition, the same pattern in relative abundance of *Streptococcus* was observed among dogs and cats with T1 resulting in a greater abundance compared to T3 and T4 (Kilburn-Kappeler et al., 2023b).

## Conclusion

The data indicate that CFP did not alter the overall diversity of the fecal microbiome of healthy adult cats over a 14-d period. The shifts in relative abundance of taxa appeared to be influenced by the type of dietary substrate available for microbial fermentation, specifically fiber and protein. In comparison to dogs, dietary treatments had a greater influence on the fecal microbiome of cats which was indicated by differences in the relative abundance of phyla as well as the increased number of genera with significant differences. A prolonged study correlating microbial shifts due to dietary intervention and overall health would be valuable in both dogs and cats. In addition, further research is warranted to determine the potential of CFP to act as a prebiotic.

## Data Availability

The data presented in this study are openly available in SRA at https://www.ncbi.nlm.nih.gov/bioproject/986920, reference number PRJNA986920.

## Abbreviations

AAFCO: Association of American Feed Control Officials
BCFA: branched chain fatty acids
BDY: brewer’s dried yeast
BW: body weight
CFP: corn fermented protein
DDGS: distillers dried grains with solubles
OTU: operational taxonomic unit
PCoA: principal coordinate analysis
SBM: soybean meal
SCFA: short-chain fatty acids
